# Investigation of Human Milk as a Biological System in a Multicenter Mother–Infant Cohort: Protocol Design and Cohort Profile of the Phoenix Study

**DOI:** 10.3390/nu16172892

**Published:** 2024-08-29

**Authors:** Jieshu Wu, Junai Gan, Guo Zeng, Xiaoqin Luo, Nianhong Yang, Zheqing Zhang, Yongye Sun, Jian Shen, Wei Wei, Jingyu Yan, Jing Zhu, Thomas Ludwig, Bernd Stahl, Xianfeng Zhao, Zhixu Wang

**Affiliations:** 1 Department of Maternal, Child and Adolescent Health, School of Public Health, Nanjing Medical University, Nanjing 211166, China; jwu@njmu.edu.cn; 2 Danone Open Science Research Center, Shanghai 201204, China; junai.gan@danone.com; 3 West China School of Public Health, Sichuan University, Chengdu 610041, China; zg_huaxi2016@126.com; 4 Department of Nutrition and Food Safety, School of Public Health, Xi’an Jiaotong University, Xi’an 710061, China; luoxiaoqin2012@mail.xjtu.edu.cn; 5 Department of Nutrition and Food Hygiene, School of Public Health, Tongji Medical College, Huazhong University of Science & Technology, Wuhan 430030, China; zynh@mails.tjmu.edu.cn; 6 Guangdong Provincial Key Laboratory of Tropical Disease Research, Department of Nutrition and Food Hygiene, School of Public Health, Southern Medical University, Guangzhou 510515, China; zzqaa501@smu.du.cn; 7 Department of Nutrition and Food Hygiene, School of Public Health, Qingdao University, Qingdao 266071, China; yongye.sun@126.com; 8 Key Laboratory of Systems Biomedicine (Ministry of Education), Shanghai Center for Systems Biomedicine, Shanghai Jiao Tong University, Shanghai 200240, China; shenjian@sjtu.edu.cn; 9 State Key Laboratory of Food Science and Resources, Jiangnan University, Wuxi 214122, China; weiw@jiangnan.edu.cn; 10 Collaborative Innovation Center of Food Safety and Quality Control in Jiangsu Province, School of Food Science and Technology, Jiangnan University, Wuxi 214122, China; 11 Key Laboratory of Separation Science for Analytical Chemistry, Dalian Institute of Chemical Physics, Chinese Academy of Sciences, Dalian 116023, China; yanjingyu@dicp.ac.cn; 12 University of Chinese Academy of Sciences, Beijing 100049, China; 13 Institute of Biotechnology and Health, Beijing Academy of Science and Technology, Beijing 100094, China; jingzhu.nutri@outlook.com; 14 Danone Global Research & Innovation Center, Uppsalalaan 12, 3584 CT Utrecht, The Netherlands; thomas.ludwig@danone.com (T.L.); bernd.stahl@danone.com (B.S.); 15 Chemical Biology and Drug Discovery, Utrecht Institute for Pharmaceutical Sciences, University of Utrecht, 3584 CS Utrecht, The Netherlands

**Keywords:** breastfeeding, human milk, infant feeding, nutrition, systems biology, HMOs, microbiome, lipidomic, proteomic, mother–infant dyads

## Abstract

Breastfeeding and human milk are the gold standard for infant feeding. Studying human milk with a systems biology approach in a large longitudinal cohort is needed to understand its complexity and health implications. The Phoenix study is a multicenter cohort study focusing on the interactions of maternal characteristics, human milk composition, infant feeding practices, and health outcomes of Chinese mothers and infants. A total of 779 mother–infant dyads were recruited from November 2021 to September 2022, and 769 mother–infant dyads were enrolled in the study. Scheduled home visits took place at 1, 4, 6, and 12 months postpartum, and 696 dyads (90.5% participants) completed the 12-month visit. At each visit, maternal and infant anthropometry was assessed. Questionnaires were administered to collect longitudinal information on maternal characteristics and lifestyle, infant feeding, and health. Digital diaries were used to record maternal dietary intake, infant feeding, and stool character. Human milk, maternal feces, infant feces, and infant saliva were collected. An external pharmaceutical-level quality assurance approach was implied to ensure the trial quality. Multi-omics techniques (including glycomics, lipidomics, proteomics, and microbiomics) and machine learning algorithms were integrated into the sample and data analysis. The protocol design of the Phoenix study provides a framework for prospective cohort studies of mother–infant dyads and will provide insights into the complex dynamics of human milk and its interplay with maternal and infant health outcomes in the Chinese population.

## 1. Introduction

The World Health Organization (WHO) recommends exclusive breastfeeding for the first six months after birth, and continued breastfeeding until two years or beyond [1]. Breastfeeding, or the provision of human milk, benefits both the infant and the mother [2,3,4].

Human milk is a complex food composed of essential nutrients and bioactive components [5]. Previous human milk composition studies provided large amounts of information, building-up human milk composition databases globally and regionally. These helped nutritionists to estimate the nutrition needs of infants [6,7,8]. As a dynamic bioactive fluid, human milk composition changes throughout the stages of lactation [9,10] and is influenced by geographic location and maternal factors, such as age, diet, lifestyle, ethnicity, and circadian rhythm [11,12,13,14]. The complexity of human milk composition underscores the need for a comprehensive understanding of its dynamic relationship with maternal and infant health at the population level. Human milk nutrients work synergistically on infant health. Recently, there has been an increased awareness that human milk is a biological system that interacts with various internal and external factors, and the current understanding of human milk composition is limited [15]. Analyzing individual components of human milk is insufficient to fully comprehend its complexity and the interplay among its diverse components [16,17,18]. Addressing this complexity requires new tools and technologies for study design and data collection [19]. Studying human milk using a systems biology approach in a large cohort is needed to decipher the intricacies of human milk and its interactions with the lactating mother and breastfed infant. This new conceptual framework has been proposed [15,19] but has not yet been well implemented.

In the recent decades, maternal–infant cohorts were initiated to explore the dynamic changes in human milk and link it to the health outcomes of infants [20,21,22,23,24]. The cohorts were designed to study the changes in human milk composition, effects of feeding practices on infant growth and development, and the extent of the interactions of infants’ gut microbiota. The Cambridge Baby Growth and Breastfeeding Study (CBGS-BF) [20] looked at profiling of breastmilk intakes and composition in relation to infancy growth in the UK. The Mothers, Infants, and Lactation Quality (MILQ) study [21,24] assessed the effects of infant feeding practices on infant growth and development in Australia. The MYBIOTA study [22] aimed at the colonization and development of the gut microbiome during early life and its impact on infant health in Malaysia. The MAINHEALTH study [23] aimed to characterize human milk from the mothers and further relate the composition to the infant gut microbiota and its metabolic impact in the infants via multi-omics analyses, including metabolomics, proteomics, glycomics, and microbiomics methods, in Denmark. The nutrition during pregnancy and early development (NuPED) study [25] was initiated to assess early nutrition-related exposures predictive of early childhood development in urban South Africa. Nevertheless, these types of cohorts still exist in limited numbers, especially in the Chinese population. 

In this paper, we describe the protocol design of the Phoenix study and characteristics of the mother–infant cohort. The objective of this study was to systematically analyze the composition of human milk utilizing multi-omics technologies, investigate longitudinal changes in infant feeding practices, and explore their impact on the health outcomes of Chinese mothers and infants. Meanwhile, maternal feces, infant feces, and infant saliva were collected for analysis, helping to capture maternal and infant phenotypes in more dimensions to further explore the relationships. Employing a multicenter observational design, we seek to understand the current need to improve maternal and infant health in China, providing a scientific basis for future interventions and solutions in this area.

## 2. Materials and Methods

### 2.1. Study Setting

The Phoenix study is a multicenter cohort study of healthy Chinese mother–infant dyads. The study was conducted at six investigative sites in six cities (Nanjing, Chengdu, Xi’an, Wuhan, Guangzhou, and Qingdao), representing the different eating habits and cuisines of East, West, Northwest, Central, South, and North areas of China. 

Screening could take place between three weeks before the expected delivery date and one month after the delivery. After informed consent and eligibility confirmation, 4 visits were scheduled for each subject from 1 to 12 months postpartum: V1 (1 month ± 1 week), V2 (4 months ± 2 weeks), V3 (6 months ± 2 weeks), and V4 (12 months ± 2 weeks). Most visits were conducted by trained researchers visiting participants at their homes, and some initial visits took place in nearby clinics or hospitals.

### 2.2. Participants and Eligibility Criteria

Healthy mother–infant dyads were recruited according to the criteria below.

Healthy mothers were eligible if they were at least 18 years old, intended to breastfeed, and provided written informed consent. Exclusion criteria for mothers included participation in other studies using investigational or marketed products concurrently or within two weeks prior to study entry; difficulty in follow-up or locating; engagement in illegal drug use, regular smoking, and/or alcohol use; body mass index (BMI) of <18.5 or ≥28 before pregnancy or before 16 weeks of gestation; presence of chronic diseases (e.g., endocrine, cardiovascular, or respiratory diseases), autoimmune diseases, psychosis and severe postpartum depression, or acute infectious diseases; presence of breast infection (e.g., mastitis, fungal infection of the nipple or areola, or reactivation of herpes simplex or varicella zoster infection in the mammary or thoracic region) at the time of enrollment; delivery of twins or multiples, or infants conceived by assisted reproductive technology (ART).

Healthy full-term infants with parents of Chinese ethnicity were eligible if they had a gestational age between 37 and 42 weeks and were breastfed at enrollment. Exclusion criteria for infants included having any congenital abnormality, chromosomal disorder, or severe disease that could interfere with study conduction and assessment, and consumption of foods other than breast milk or infant formula.

### 2.3. Data Collection

During the study visits, trained researchers measured the anthropometrics of mothers and infants, interviewed mothers with a set of questionnaires, and collected samples from the subjects. In addition, mothers were provided with self-administered digital diaries to record maternal dietary intake, infant feeding, and infant stool frequency and consistency. The schedule of these activities is shown in Table 1.

#### 2.3.1. Anthropometric Assessment

Anthropometric measurements were taken by trained researchers using a standardized procedure. Maternal anthropometric measurements included weight and height. Maternal body weight (in kilograms) was measured to the nearest 0.1 kg using a calibrated weighing scale (Yiqing, Senssun, Zhongshan, China) without wearing shoes or heavy clothing. Maternal height (in centimeters) was measured to the nearest 0.1 cm. Infant weight (in grams) and length (in centimeters) were measured using a digital baby scale (iR-Baby, Senssun, Zhongshan, China) with a resolution of 0.1 cm and 5 g, respectively. Head circumference (in centimeters) of the infant was measured as the distance over the most prominent point on the back of the head (occiput) and just above the eyebrows (supraorbital ridges) using a flexible, non-stretchable measuring tape (Wentai, Foshan, China) to the nearest 0.1 cm by trained researchers.

#### 2.3.2. Questionnaires

A set of questionnaires were developed for data collection in this study. These questionnaires were either administered through face-to-face interviews with the subject by a trained researcher or completed by the subject under the guidance of the researcher.

- Sociodemographic information, maternity, and infant birth

At V1, a general information questionnaire was used to collect the following information: (1) sociodemographic information, covering parental characteristics (date of birth, sub-ethnicity, marital status, educational level, occupational status, average working hours, etc.), as well as household characteristics (household size, annual income, type of housing, energy usage in kitchen or for heating, use of disinfectants, presence of pets, maternal and infant exposure to second-hand smoke, etc.); (2) maternity information, consisting of gravidity, parity, weight, last menstrual date, expected due date, pregnancy complications, pre-pregnancy medical history, and breastfeeding experience; (3) infant birth information, including date of birth, gestational age, delivery mode, sex, birth weight, birth length, birth head circumference, health problems at birth, first food after birth, etc. Information was verified through medical records or birth certificates when available.

- Maternal dietary intake

Maternal dietary intake was assessed at each visit using the Food Frequency Questionnaire (FFQ) developed in this study. The FFQ consisted of food items that were grouped into the following categories: cereals, legumes, meat, fish, dairy, fungi, vegetables, fruits, nuts, snacks, beverages, cooking oil, and nutritional supplements. For each item, the participants were asked to recall their usual frequency of intake and portion size during the past month with the help of a food atlas [26]. Maternal food consumption, including frequency and portion size, were evaluated.

- Maternal physical activity and sleep

The maternal physical activity and sleep questionnaire was developed based on the International Physical Activity Questionnaire (IPAQ) [27] and the Pittsburgh Sleep Quality Index (PSQI) [28], with slight modifications, to assess maternal physical activity (including vigorous-intensity activities, moderate-intensity activities, walking, and sitting) and sleep (duration and quality) over the past week.

- Maternal and infant diseases

Infections and other diagnosed illnesses, from delivery or from the previous visit to the current visit, of the mother and the infant were recorded based on medical records or by self-report. Medication use as well as the start and end dates of symptoms were recorded.

- Infant skin and respiratory symptoms

Skin and respiratory symptoms were evaluated for infants during each visit. The assessment of skin symptoms included the presence or absence of rashes and the area and severity of rashes. The assessment of respiratory symptoms involved the severity of choric cough, runny nose, and asthma. The intention of these questions was not the diagnosis of allergy, but to identify infants with an increased risk or likely diagnosis of allergy.

- Pediatric quality of life

The Pediatric Quality of Life Inventory (PedsQL^TM^) Infant Scales [29] was used for the assessment of the quality of life of infants aged 1 to 12 months. The parents’ report for infants of the PedsQL^TM^ consists of 36 items on 5 dimensions (physical functioning, physical symptoms, emotional functioning, social functioning, and cognitive functioning).

- Infant Gastrointestinal Symptom Questionnaire

The Infant Gastrointestinal Symptom Questionnaire (IGSQ) [30], a 13-item interviewer-administered questionnaire, was used to assess the gastrointestinal tolerance of infants over the past 7 days. The questionnaire contains 13 questions grouped into 5 categories (stooling, spitting up/vomiting, crying, fussiness, and flatulence) that describe the infant’s gastrointestinal status, from no gastrointestinal distress to extreme gastrointestinal distress.

- Infant feeding practice

Infant feeding behaviors, such as type of feeding (direct breastfeeding, expressed breastmilk feeding, formula feeding, and mixed-milk feeding), frequency of feeding in the past week and past month, and timing of first food introduction, were recorded based on maternal recall. In addition, maternal feeding experiences during the past month were documented. 

#### 2.3.3. Digital Diaries

Digital services were developed to record maternal dietary intake, infant feeding, and infant stool diaries. Instructions were provided so that the mothers could record the diaries on their smartphones. Mothers recorded their dietary intake (including food and the estimated amount) and their infants’ feeding (including the frequency and intake/duration of the feeding) for 24 h with a validated digital service tool based on the Chinese Food Composition database [31]. In addition, mothers were asked to upload the stool images and record the stool frequency and consistency of their infants for five days. The stool consistency was further assessed by a validated AI-based algorithm according to the Brussels Infant and Toddler Stool Scale (BITSS) [32]. 

#### 2.3.4. Sample Collection

Biological samples, including human milk, maternal feces, infant feces, and infant saliva, were obtained by non-invasive procedures. Instructions for sample collection were provided in videos and handouts for researchers and subjects. Materials needed for sample collection were prepared at the central lab (APTBIO, Shanghai, China) before shipping to the sites. The samples were collected by research stuffs and kept in an icebox immediately. Then, all of them were express shipped to the study site’s lab within 10 h in an icebox and stored at −80 °C before periodic shipping to the central lab on dry ice.

- Human milk collection

Human milk collection was conducted at each visit unless the subject had stopped lactation. Mothers were asked to avoid applying skincare or any other agents to the breast 24 h prior to the sample collection. Fresh human milk was collected from one breast in the morning (between 9 a.m. and 11 a.m.) after 2 h since the last feed/expression from that breast. Mothers were instructed to clean the breast with sterile saline and gauze pads, and fully express fore- and hind-milk aseptically using a hospital-grade electric breast pump (Symphony, Medela AG, Baar, Switzerland) equipped with sterile single-use accessories (008-0276, Medela AG, Baar, Switzerland). The total volume of milk from this full expression was recorded. After being gently mixed, a volume of approximately 40 mL was collected into six tubes (15 mL tubes—Corning, Shanghai, China; 10 mL tubes—Titan, Shanghai, China; 5 mL tubes—Jiete, Guangzhou, China) for the study and the remaining milk was returned to the subject for infant feeding. Participants who expressed no more than 40 mL of human milk were also accepted for continue, and nobody dropped out of the study for this reason.

- Maternal fecal sample collection

The maternal fecal sample was collected at the first visit. A stool collection kit and instruction materials were given to mothers. Mothers were asked to urinate before stool collection to avoid urine contamination. A disposable stool box (GWF01, TinyGen, Shanghai, China) was used to hold the stool. The specimen was taken from the middle of the stool and put into the fecal sample container (LAF25, Biorise, Shanghai, China).

- Infant fecal sample collection

Infant fecal sample collection was conducted at each visit. A fraction of feces was collected from a concentrated portion of feces from a diaper and put into a fecal sample container (LAF25, Biorise, Shanghai, China).

- Infant saliva collection

Infant saliva was collected by trained researchers in the morning during the study visit (V2), at least 60 min after the last feeding. A saliva oral swab (Oracol S10, Malvern Medical Developments, Worcester, UK or Salivette, Sarstedt, Nümbrecht, Germany) was placed at or moved around the corner of the mouth for 1 to 2 min for saliva collection. 

### 2.4. Laboratory Analysis on Biological Samples

In-depth analyses of biological samples, including multi-omics approaches, was performed in designated specialized analytical laboratories. Human milk samples were analyzed for macronutrients (total carbohydrates, total protein, total fat, and total energy level), micronutrients (including vitamins, i.e., vitamin A, thiamin, riboflavin, folate, vitamin C, vitamin E, etc., and minerals, i.e., calcium, iron, magnesium, selenium, zinc, etc.), and bioactive components (including oligosaccharides, amino acids, proteome, lipidome, and microbiome). The content of carbohydrates in human milk was measured using the DNS total carbohydrate assay kit, total lipids was measured using the Röse–Gottlieb method, and total protein was measured using the BCA protein assay kit. The total energy level was calculated from the macronutrient levels. Vitamins were analyzed by liquid chromatography coupled to mass spectrometry (LC-MS), and minerals were analyzed by inductively coupled plasma mass spectrometry (ICP-MS). Human milk oligosaccharides (HMOs) were measured by LC-MS. Amino acids and protein profiles were analyzed by LC-MS. Regarding human milk fat, fatty acid composition and *sn*-2 fatty acid positional distribution were analyzed by gas chromatography (GC), while triacylglycerol profile and polar lipids were analyzed by LC-MS. The human milk microbiome, maternal gut microbiome, and infant gut microbiome were analyzed by 16S rRNA gene sequencing of the V3–V4 hypervariable region, and metagenomic analysis was performed on a subset of these samples. Infant saliva samples were analyzed by the enzyme-linked immunosorbent assay (ELISA) to determine the Lewis and Secretor phenotypes.

### 2.5. Data Management and Monitoring

**Figure 1:**
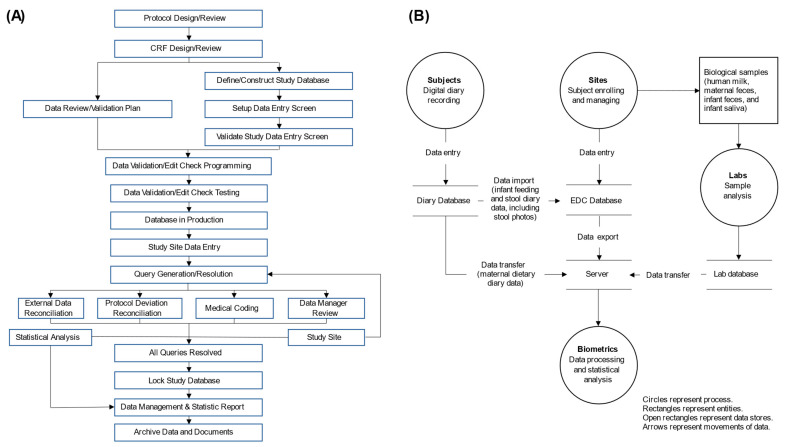
Data management workflow (**A**) and data flow diagram (**B**).

A data management plan was developed to define the key data management processes, procedures, and rules for collecting, entering, storing, managing, reviewing, cleaning, and reporting of all data in this study (including, but not limited to, EDC data, external lab data, and other external data). Data management and data flow diagrams are shown in Figure 1. Viedoc, a Clinical Data Interchange Standards Consortium (CDISC)-compliant electronic data capture (EDC) system, was used for capturing and managing the study data. Data entry was performed by authorized site researchers. Both on-site and centralized monitoring were conducted by Clinical Research Associates (CRA). Data review was performed by the data manager (DM) from the contract research organization (CRO). 

### 2.6. Quality Control

All site staff (researchers) received training on Good Clinical Practice (GCP) guidelines. Researchers were trained on the activities in which they were involved and given authorization before carrying out the activities. Source data verification (SDV) of 100% site data entered to the EDC system was performed by CRA. Quality check co-monitoring visits and quality audits were performed to ensure the quality of the study. To ensure sample quality, temperature was monitored during sample storage and shipment. The ice in the icebox was checked by researchers and recorded in sample transportation form. Sample information was verified by the receiving laboratories on each shipment, and all sample checks were documented. Method validation was required prior to sample testing, and quality-control measures were implemented during sample testing, such as a blank/negative control for each batch of extractions in all the tests.

### 2.7. Statistical Analysis

Descriptive analysis was performed to describe the sociodemographic and clinical characteristics of the participants and to summarize the results of the questionnaires. Normality of the continuous data was tested, and transformations were applied if needed. Continuous variables were compared between groups using a one-way analysis of variance or Student’s *t* test, and categorical variables were compared using the Pearson Chi-square test or Wilcoxon rank-sum test. A two-tailed *p*-value ≤ 0.05 was considered statistically significant.

Images collected from digital diaries were classified using a convolutional neural network algorithm. Omics data files were processed by specialized software to obtain the absolute concentration or relative abundance of the detected molecules, and machine learning methods were applied to multi-omics integration to investigate the relationship between these data and maternal and infant health.

For the cross-sectional data analysis, logistic or linear regression was used when the outcome was binary or continuous, respectively. For the longitudinal data analysis, linear mixed-effect models or multilevel mixed-effect models were performed after adjusting for time-dependent and static covariates. Potential confounders, such as age, gender, and BMI, were evaluated during data analysis. Interactions among potentially influencing factors were tested for potential effect modification using multivariate analysis. 

## 3. Results

**Figure 2:**
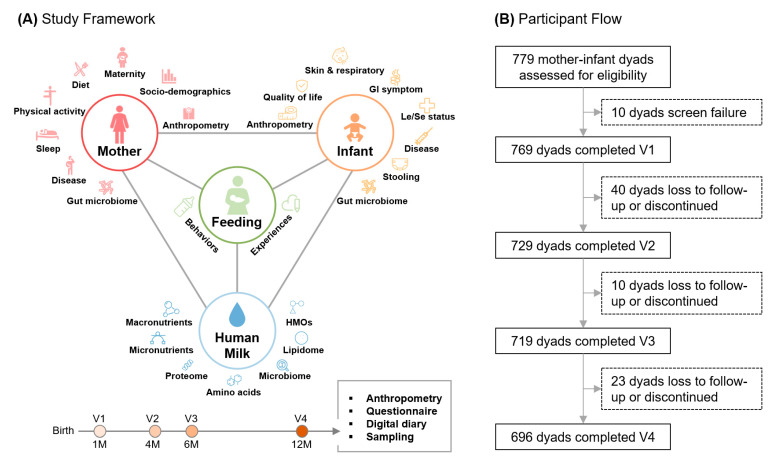
Study framework (**A**) and flow chart of participants (**B**).

In this study, the characteristics and health status of mother–infant dyads were systematically assessed longitudinally by anthropometric assessment, questionnaire survey, digital diary, biological sampling, and subsequent multi-omics analyses (Figure 2A). From November 2021 to September 2022, 779 mother–infant dyads were screened at 6 sites. Screening failures included withdrawal or loss of contact prior to the confirmation of eligibility at V1 (*n* = 6), difficulty in follow-up or locating (*n* = 2), presence of chronic disease (*n* = 1), and infant not breastfed at enrollment (*n* = 1). A total of 769 mother–infant dyads were enrolled and completed the 1-month visit. Study retention was 94.8% (729 dyads), 93.5% (719 dyads), and 90.5% (696 dyads) at the 4-month, 6-month, and 12-month visits, respectively (Figure 2B). Other participants dropped out due to leaving the city or it being inconvenient to visit within the follow-up time.

Maternal and infant characteristics are summarized in Table 2. Maternal age ranged from 19 to 44 years, with a mean of 31.1 years. All infants were term born at a gestational age between 37 and 42 weeks, with a mean of 39.4 weeks.

## 4. Discussion

This study provided a framework for a prospective cohort study of mother–infant dyads with the concept of systems biology embedded in the design. Considering that maternal diet and lifestyle may vary across different regions in China, which may affect infant feeding and health outcomes, we employed the multicenter observational design to capture the geographical variation. Integration of new technologies, such as omics techniques and artificial intelligence, supports data collection and analysis [19,33]. In this study, the characteristics and health status of mother–infant dyads were captured and analyzed using a variety of approaches, including anthropometric assessment, questionnaire survey, and digital diary recording with AI-based assessment. Along with the extensive metadata, biological samples including human milk and fecal samples, will be analyzed using state-of-the-art multi-omics techniques. Extensive omics data, such as HMOs, lipidome, proteome, and microbiome, will be generated. Machine learning algorithms will be integrated into the data analysis. This is the most comprehensive multicenter cohort study utilizing various approaches and trending methods to investigate human milk and mother–infant dyads in China. In-depth analysis of this comprehensive dataset with a multidisciplinary team approach will help us better understand the complexity of human milk and feeding practices and their interactions with the mother and infant.

The dropout rate at the 12-month visit in this study was 9.5%, which is low compared to similar longitudinal trials. To ensure participant compliance and adherence, we chose to conduct most of the visits in participants’ homes rather than in hospitals, as home visits are more convenient for mothers and infants. Home visits also help improve the accuracy of the information collected. For example, mothers could easily assess their medical records at home to verify the information, and the researchers can also verify the food portions by investigating the subjects’ dining bowls. Efforts were made to ensure the quality of this large cohort study. All the researchers in this study needed to be trained before any home visits, and their training logs were strictly reviewed. Sample collection and handling procedures were designed and reviewed, and instructions in videos and handouts were prepared in advance. Since human milk composition varies within a feed [34], a full expression of foremilk and hindmilk was collected. When the human milk microbiome is targeted for analysis, manual expression is usually performed to avoid contamination from the pump [35,36]. To our knowledge, this is the first large cohort study to use a hospital-grade electric breast pump equipped with sterile single-use accessories to aseptically collect a full expression of human milk, allowing for accurate measurements of milk components, including nutrients and microbiome. 

Meanwhile, human milk is complex and interacts with multiple factors. Human milk composition varies with lactating time and circadian rhythm. The quality of sample collection and handling also significantly impacts the potential read-outs. We implemented strict quality control throughout the study, including well-trained researchers, strict sample collection times, and a well-designed collection protocol, sample transportation protocol, and handling record. To ensure the quality of the study, we implemented quality monitoring and assurance system from a pharmaceutical standard based Contract Research Organization (CRO, ClinChoice (Beijing) Medical Science Technology Ltd, Beijing, China) compliance to the International Council for Harmonization (ICH) guidelines for good clinical practice (GCP), and sample checking by the central lab (APTBIO, Shanghai, China). 

Currently, site data entered into the EDC system have been verified, and results related to the anthropometric assessment, questionnaires, and digital diaries will start to come out this year. Laboratory analysis on biological samples is ongoing—sample analysis results as well as their correlations with health outcomes are expected in the next two to three years.

This study has several limitations. First, the study focused on the Chinese ethnicity, which may limit the generalizability of the findings to other populations. Second, the infant health outcomes examined in this study are related to growth, gut health, and immunity, while cognitive and other aspects were not examined. Further follow-up is planned to look at child growth and development in more areas, including but not limited to cognitive development, etc.

## 5. Conclusions

The paper described the design and protocol of a multicenter longitudinal study investigating the dynamics of human milk composition, infant feeding practices, and their impact on the health outcomes of Chinese mothers and infants. The findings will provide insights into the intricate nature of human milk and its interplay with maternal and infant well-being, potentially shaping future strategies for promoting their health.

## Figures and Tables

**Table 1:** Schedule of anthropometric assessment, questionnaire interview, digital diary recording, and sample collection in the study.

		V1	V2	V3	V4
		1 Month ± 1 Week	4 Months ± 2 Weeks	6 Months ± 2 Weeks	12 Months ± 2 Weeks
Anthropometric assessment	Maternal height	√			
	Maternal weight	√	√	√	√
	Infant weight, length, and head circumference	√	√	√	√
Questionnaires	Sociodemographic, maternity, and infant birth	√			
	Maternal dietary intake (FFQ)	√	√	√	√
	Maternal physical activity and sleep	√	√	√	√
	Maternal and infant diseases	√	√	√	√
	Infant skin and respiratory symptoms	√	√	√	√
	Pediatric quality of life (PedsQL)	√	√	√	√
	Infant gastrointestinal symptom questionnaire (IGSQ)	√	√	√	√
	Infant feeding practice	√	√	√	√
Digital diaries	24 h maternal dietary intake diary	√	√	√	√
	24 h infant feeding diary	√	√	√	√
	5-day infant stool frequency and consistency diary	√	√	√	√
Sample collection	Human milk sample	√	√	√	√
	Maternal fecal sample	√			
	Infant fecal sample	√	√	√	√
	Infant saliva sample		√		

**Table 2:** Characteristics of the study participants.

Characteristics	Mean ± SD/*n* (%)
Mothers (*n* = 769)	
Age, years	31.1 ± 3.8
Ethnic groups	
Han	754 (98.0%)
Zhuang	2 (0.3%)
Hui	9 (1.2%)
Other	4 (0.5%)
Education	
Below primary school	1 (0.1%)
Primary school	2 (0.3%)
Junior high school	42 (5.5%)
Senior high school	51 (6.6%)
Junior college	194 (25.2%)
Bachelor	344 (44.7%)
Postgraduate or above	135 (17.6%)
Occupational status	
Employed	557 (72.4%)
Self-employed	55 (7.2%)
Housewife	120 (15.6%)
Student	5 (0.6%)
Other	32 (4.2%)
Infants (*n* = 769)	
Gestational age, weeks	39.4 ± 1.0
Birth weight, g	3340.4 ± 372.9
Sex	
Male	390 (50.7%)
Female	379 (49.3%)
Delivery mode	
Vaginal delivery	432 (56.2%)
Caesarean section	337 (43.8%)

## Data Availability

The original contributions presented in the study are included in the article, further inquiries can be directed to the corresponding author.
